# The subjective and objective quality of life score (SOQOLTM) for the quantification of general health status: a preliminary study with initial normative population values

**DOI:** 10.1186/s41687-024-00684-1

**Published:** 2024-01-18

**Authors:** Ralph J. Mobbs, R. Dineth Fonseka, Pragadesh Natarajan, Lianne Koinis, Monish Maharaj

**Affiliations:** 1 NeuroSpine Surgery Research Group (NSURG), Sydney, Australia; 2 NeuroSpine Clinic, Prince of Wales Private Hospital, Randwick, Australia; 3 Faculty of Medicine, University of New South Wales, Sydney, Australia; 4 Wearables and Gait Assessment Research Group (WAGAR), Sydney, Australia; 5 Prince of Wales Private Hospital, Suite 7, Level 7, Randwick, NSW 2031 Australia

**Keywords:** Walking, Gait analysis, Walking speed, Gait speed, Step count, Daily step count, Objective, Health metrics, Gait metrics, QOL, SOQOL, SMoS, Pain, Depression, Anxiety

## Abstract

**Background:**

Established health-related quality of life scores do not consider both subjective and objective indices of health. We propose the subjective and objective quality of life score (SOQOL) for the comprehensive assessment of health-related quality of life and aim to provide normative population data. The SOQOL is compatible with smartphone applications, allowing widespread use on a global scale.

**Methods:**

Normative SOQOL population data was sourced from pre-existing datasets on the EQ-5D-5L, daily step count, and walking speed. Normative values were calculated using weighted grand means. We trialled the SOQOL in a group of five patients presenting to a spinal neurosurgery clinic.

**Results:**

SOQOL scores decreased with age, and women had lower scores in every age group. In our case series, the spine patients with the biggest SOQOL deficit compared to age- and sex-matched population averages were found to be surgical while the rest were non-surgical.

**Conclusions:**

The SOQOL shows promise as a simple and effective scoring tool that is compatible with smartphones, potentially useful for screening in primary and specialized care settings, and for assessment following healthcare interventions. This study, however, is preliminary, and the findings are primarily suggestive. They underline the necessity for future, more comprehensive studies to validate and expand upon these initial observations. The conclusion of both this abstract and the full paper will clearly state these limitations and the preliminary nature of the study.

## Introduction

The World Health Organisation defines quality of life (QOL) as “an individual’s perception of their position in life in the context of the culture and value systems in which they live and in relation to their goals, expectations, standards and concerns" [1]. In the setting of health care, this can be measured using a variety of indices such as physical health, mental health, social health, and functional health.

A variety of scoring tools have been proposed to measure health-related QOL. Of note are the EQ-5D [2] (EQ-5D) and the short form health survey (SF-36) [3] which have been validated across a variety of disease states and populations [4–9]. Other scoring tools are disease- or specialty-specific, such as the Oswestry Disability Index for low back pain [10], and are not suitable as general health measures. Our research group recently proposed a health-related QOL score called the Simplified Mobility Score (SMoS™) [11] which instead uses objective metrics (daily step count and average walking speed) to provide insight into a person’s mobility health. Although these are activity metrics, they are relevant in a wide variety of disease states, including but not limited to neurological [12], musculoskeletal [13], and psychiatric [14] illnesses. However, the SMoS does not account for the patient’s own perception of their disease. As the majority of QOL instruments are by design completed by patients or caregivers, they provide a subjective assessment of health. To our knowledge, there is no health-related QOL scoring tool which combines both subjective and objective metrics of health.

Recently, there has been developing interest in the incorporation of smartphones into health care assessment. As of 2023, the global population continues to see increases in smartphone uptake, as suggested by the number of smartphone mobile network subscriptions increasing from 3.6 billion in 2016 to 6.7 billion in 2023 [15]—forming a majority of the world’s population. It follows that a health-related quality of life scoring tool which is compatible with smartphones can be implemented on a global scale. In addition, smartphones, or wearable devices more generally, can allow remote monitoring, where health care providers can be provided with information about their patients without having to meet in-person. This allows for continuous patient monitoring.

In this paper, we propose a general health-related QOL score, the subjective and objective quality of life score (SOQOL), adapting elements of the EQ-5D-5L and SMoS™. We aim to compile population normative data using pre-existing datasets and trial the SOQOL in a small cohort of patients.

## Methods

### Calculation of SOQOL

**Fig. 1:**
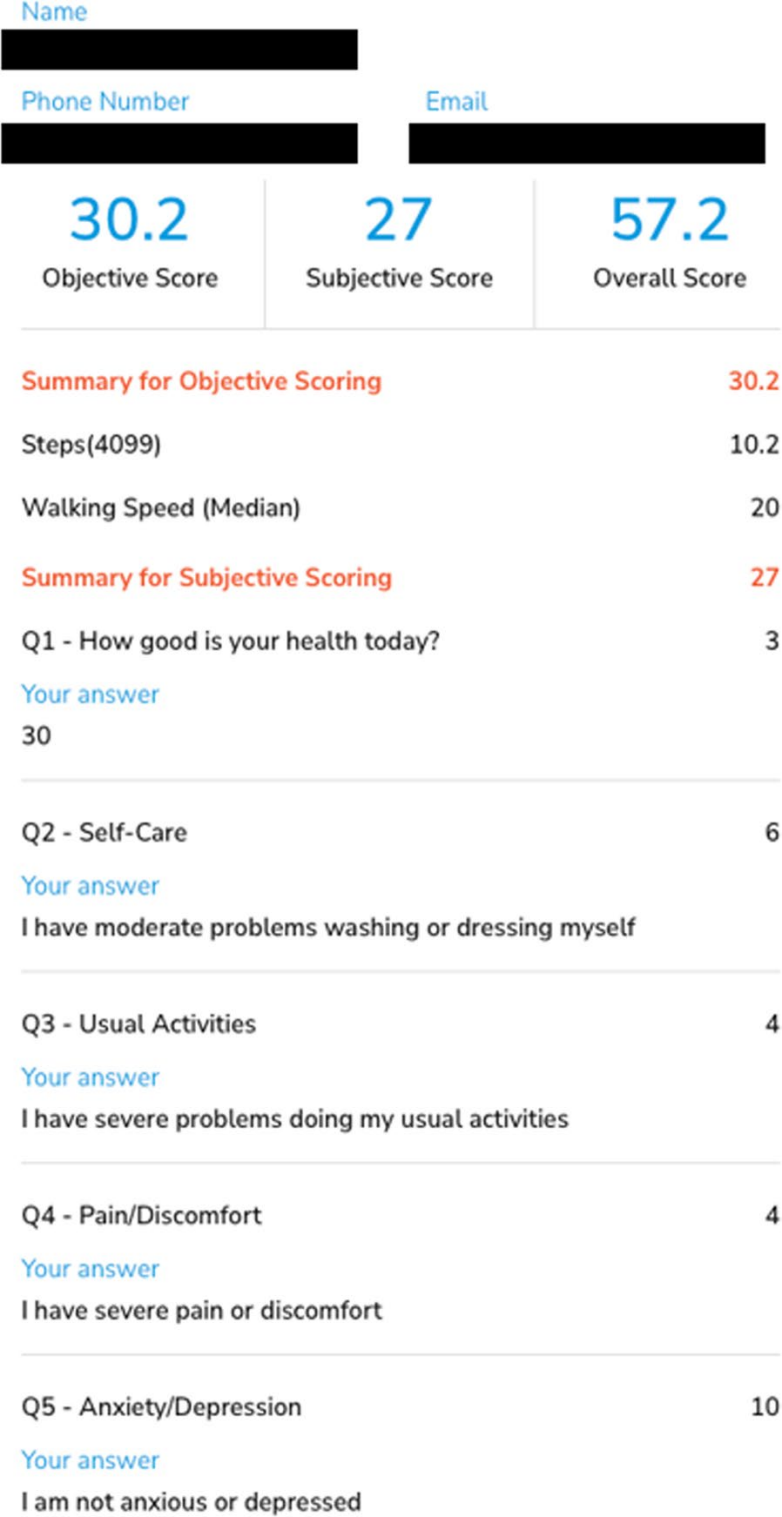
Measurement of the subjective component of the SOQOL using an example from one of the patients in the present study. Each of the questions are initially presented to the user on separate pages

We propose a simple metric—the subjective and objective quality of life score (SOQOL) which is measurable with smartphones and combines objective and subjective measures of health. The subjective component draws inspiration from the generic, widely used, and well-validated EQ-5D-5L score and contains five self-rated items—(1) overall impression of health, (2) personal care, (3) day-to-day activity, (4) pain and discomfort, and (5) anxiety and depression. Each item is scored out of 10, resulting in a maximum total of 50 points for the subjective component of SOQOL. The objective component of SOQOL is adapted from the SMoS, which incorporates walking speed and daily step count, each scored out of 25, forming a total of 50 points. Together, the subjective and objective components of SOQOL form a maximum of 100 points, where higher values correlate to better general health status. This is summarized in Fig. 1 and Table 1.

**Table 1:** Calculation of the objective component of the SOQOL

Walking speed (WS)		Daily step count (DSC)	
WS (*v*)	Points (A)	DSC	Points (B)
*v* < 1.35 m/s	(*v*/1.35) × 25	DSC < 10,000	(DSC/10,000) × 25
*v* > 1.35 m/s	25	DSC > 10,000	25

Objective component of SOQOL = A + B

### Data collection

Data for the subjective component of this score was sourced from large population studies collated by EuroQol on their website under the “Population Norms” subheading [2]. Studies were included if they contained age group data on any of the questionnaire items of the EQ-5D-5L.

Data for the objective component of this score was sourced from a large population meta-analysis measuring walking speed (n = 23,111) [16] and the worldwide Argus dataset for daily step count (n = 717,527) [17]. Further information can be found in the source publications.

**Fig. 2:**
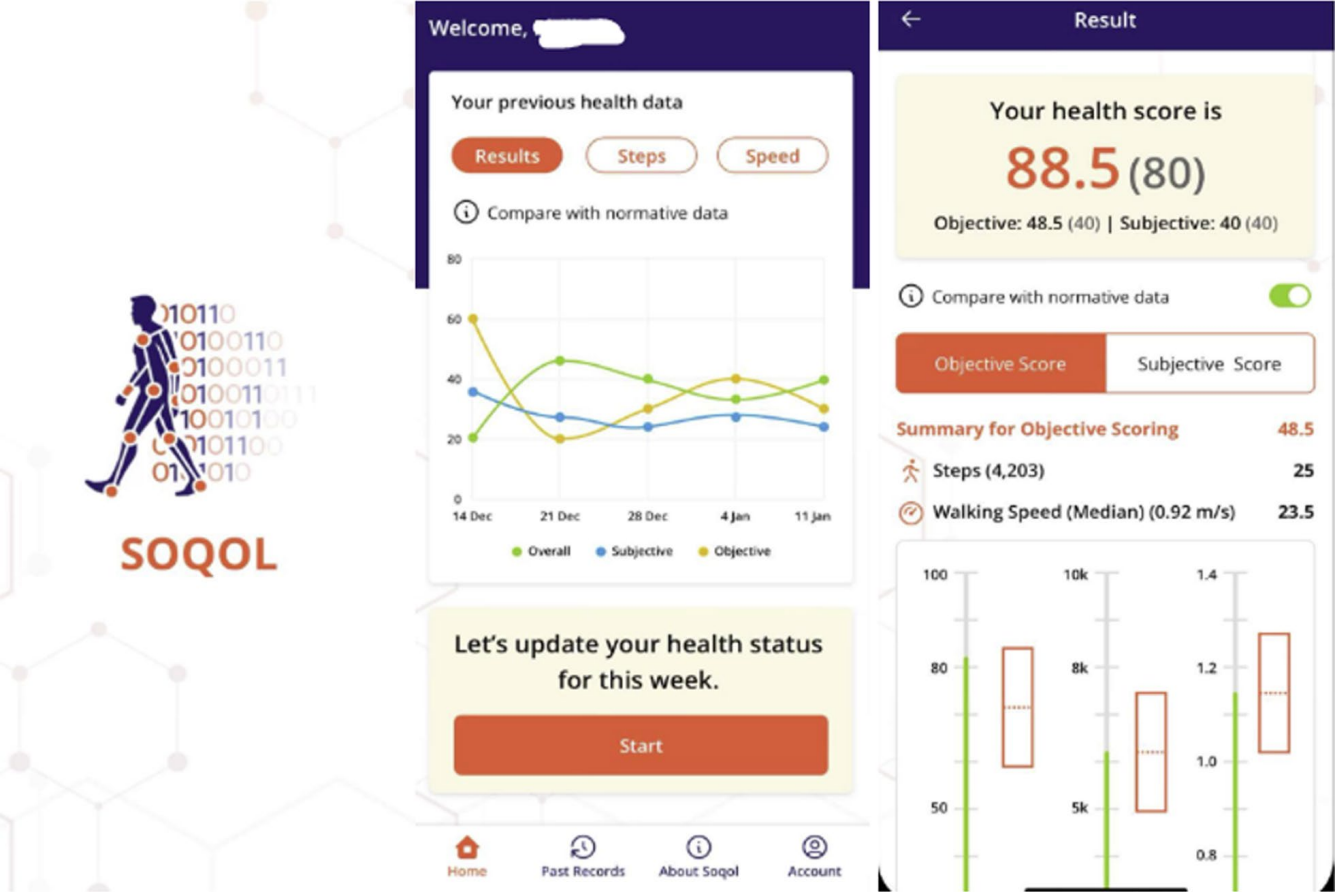
Screenshots from the smartphone based SOQOL application

We trialled the SOQOL in a spinal neurosurgery clinic at Prince of Wales Hospital, Sydney, Australia, between July and November of 2022 in five randomly selected patients. Each patient was consented to the study and downloaded onto their personal smartphone an application that we built. Patients could only be included if they had an iOS smartphone as the application is currently only available on the Apple App Store. Screenshots of the application are shown in Fig. 2. Patients were followed-up for the duration of the study and were asked to update data for the subjective component for SOQOL each week. Objective data was captured using in-built activity data from each patient’s smartphone and weekly averages were taken. Data was uploaded from the application onto an encrypted website from which we could analyse the data. Patients were classified as surgical or non-surgical depending on whether they had undergone or were planned to undergo surgery as of February 2023.

### Data analysis

Formal mean comparison statistical tests were not appropriate as the included normative papers did not provide data about the variance of the samples by age group and only provided mean values and sample size. Instead, the weighted grand mean was presented for each age bracket by sex. This is calculated as shown below:

$$\text{Weighted grand mean } = \sum \frac{{x}_{i}}{n} ,$$

where *x*_*i*_ = the *i*th observation in the total dataset with all the included studies, and *n* = the total number of observations in the dataset with all the included studies.

## Results

### Demographic data

Data from 194,902 men and 169,197 women were eligible for analysis of normative data. Most male participants (n = 54,067) were from the 30 to 39-year-old age group. Most female participants (n = 49,016) were from the 20 to 29-year-old age group.

Seven of the studies collated by EuroQol were included in our normative dataset. These are summarised in Table 2.

**Table 2:** Demographic data from EuroQol normative dataset

Author	Population sampled
Poder et al. [18]	Quebec
Yang et al. [19]	China
Jensen et al. [20]	Denmark
Marten et al. [21]	Germany
Shiroiwa et al. [22]	Japan
Garratt et al. [23]	Norway
Teni et al. [24]	Sweden

Of the five patients trialling the SOQOL score, four were male and one was female. Their demographic data is shown in Table 3.

**Table 3:** Demographic data of recruited patients

Patient ID	Sex	Age	Primary diagnosis	Surgical or non-surgical
1	M	54	Intervertebral disc degeneration	Surgical
2	M	65	Facetogenic and/or discogenic low back pain	Surgical
3	M	81	Facetogenic and/or discogenic low back pain	Non-surgical
4	M	67	Lumbar spinal stenosis	Non-surgical
5	F	48	Lumbar disc herniation	Non-surgical

### Population SOQOL values

Formal quantitative statistical tests were not appropriate as the selected papers did not provide data about the variance of the samples by age group and only provided mean values and sample size.

**Fig. 3:**
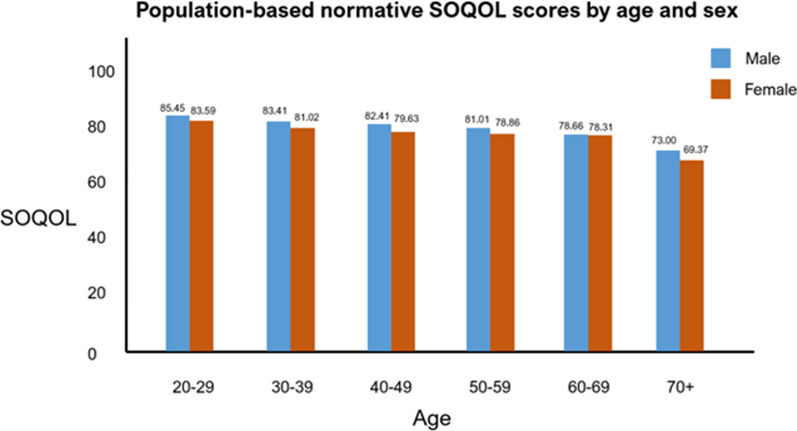
Normative SOQOL score by age bracket and sex

We calculated mean SOQOL scores by age group and sex using the grand weighted mean. These results are summarised in Fig. 3. For each age bracket, this represents the sum of each data point divided by the joint sample size, such that studies with a greater sample size will weigh more heavily on the mean. The mean SOQOL score by age group for men ranged from 85.45 for the 20–29-year-old group to 73.00 for the 70+ year-old group. The mean SOQOL score for age group for women ranged from 83.59 for the 20–29-year-old group to 69.37 for the 70+ year-old group. Table 1 and Fig. 1 display population means of SOQOL scores by age group for men and women.

For both sexes, the mean SOQOL score decreases by 1–3-point increments for each 10-year age group until age group 60–69, where there was then a steep decrease by 5.66 points for men and 8.94 points for women to the 70+ age group. Across all age groups, males had consistently higher SOQOL scores by 2–4-point increments.

### Comparison between population SOQOL values and recruited patients

As shown in Tables 4 and 5, four out of five patients had lower SOQOL scores compared to their age- and sex-matched population average. The two patients with the biggest deficit between their SOQOL score and the age- and sex-matched average were found to be surgical, with the other patients being non-surgical.

**Table 4:** SOQOL data of recruited patients

Patient ID	Q1	Q2	Q3	Q4	Q5	Walking speed	Daily step count	SOQOL	Age- and sex-matched SOQOL
1*	3	6	4	4	10	20	10.2	57.2	81.01
2*	3	8	6	4	6	23.1	12.3	62.4	78.66
3	7	10	8	8	10	15.9	2.2	61.1	73.00
4	2	6	4	4	8	22.4	22.4	68.8	78.66
5	0	10	10	10	10	22.4	21.2	83.6	79.63

Q1-5 refer to the items in the subjective component of SOQOL, as per Fig. 1

*Surgical patients

**Table 5:** SOQOL normative data by age bracket and sex

	n	Subjective component	Objective component	SOQOL
(a) Men				
20–29	42,696	46.99	38.46	85.45
30–39	54,067	45.46	37.94	83.41
40–49	42,770	44.87	37.54	82.41
50–59	25,701	44.17	36.84	81.01
60–69	15,189	44.41	34.25	78.66
70+	14,479	42.78	30.22	73.00
(b) Women				
20–29	49,016	46.75	36.84	83.59
30–39	36,801	44.18	35.62	81.02
40–49	26,560	44.02	35.43	79.63
50–59	20,679	43.43	34.28	78.86
60–69	16,081	44.04	32.00	78.31
70+	20,060	41.64	27.74	69.37

## Discussion

SOQOL is potentially compatible with smartphone-based data capture, which could allow for the measurement of health-related quality of life data on a global scale. We propose SOQOL as a comprehensive QOL tool that takes into account both subjective and objective markers of health. In this study, we pooled population normative SOQOL scores by age and sex to provide a baseline against which pathological patient groups can be compared. We also trialled the SOQOL in a small cohort of lumbar spine patients and found that most had lower SOQOL scores compared to population averages. A remote monitoring platform leveraging SOQOL may be able to provide clinicians with real-time data including but not limited to disease severity, the effectiveness of interventions based on pre- and post-intervention scores, and trends in health status that allow early identification of new disease states.

We found that normative SOQOL scores decreased with increasing age and were lower across all age brackets in women compared with men. The relatively lower subjective scores in women may be because, in many sociocultural contexts, men perceive an expectation to internalise concerns, and therefore may be less likely to report poor health outcomes in a questionnaire, as suggested by Sorensen et al. [25]. The differences in subjective scores between men and women may also be because of the difference in mental health outcomes between the two sexes. For example, Kessler [26] performed a review of population studies comparing mental health outcomes between men and women and estimated that 29% of women expression major depressive disorder in their lifetime compared to 18% of men. On the other hand, the difference in the objective component of SOQOL between men and women may reflect an increased prevalence of physical inactivity in women compared to men. Guthold et al. [27] pooled data from population surveys on physical activity and reported that a higher proportion (28.6–39.0%) of women compared to men (21.1–30.7%) were not able to meet at least 150 min of moderate-intensity, or 75 min of vigorous-intensity physical activity per week. This would explain the discrepancy in daily step count between the two sexes. Meanwhile, a difference in average walking speed between the two sexes may not truly reflect different health outcomes; for example, women are on average of a shorter height than men, and hence will have a shorter step length, and hence slower walking speed. Future studies analysing the difference in SOQOL scores between men and women may inform public health interventions aimed at reducing health inequality.

In our study, four out of five patients had lower SOQOL scores compared to age- and sex-matched population averages. This is unsurprising, as all presented with lumbar spine pathology. Although this was a small sample size, it is interesting to note that the patients with the biggest deficit compared to normative values were the two who were later classified as surgical patients. This suggests that SOQOL has differentiating power between varying disease severity, from those requiring conservative management to those requiring more intensive management. We would expect this to be the case, given that both EQ-5D and SMoS have shown differentiating ability between varying disease severity. For example, a review published by Dyer, Goldsmith, Sharples and Buxton [28, 29] found that EQ-5D scores decreased from a mean of 0.78 to 0.51 for mild to severe disease in heart failure patients. Moreover, mean SMoS score for non-operative spine patients was 62.1 compared to 50.2 for operative patients (*p* < 0.05) [11]. However, before SOQOL can be regarded as having differentiating ability between varying disease severity, studies with larger sample sizes that are better representative of different age, sex, and disease groups are required.

Scoring tools like SOQOL may also be used to assess the effectiveness of interventions, by measuring the health-related QOL of an individual at timepoints before and after their intervention. An effective intervention would result in an improvement in health-related QOL. This is important both for health care providers to confidently recommend interventions to patients, and for hospitals and insurance providers to be satisfied with the costs associated with interventions. However, to our knowledge, there are no other health-related quality of life scores in the literature which incorporate both subjective and objective measures of health. Both components are important. A subjective component is important to preserve patient-centred care and convey the patient’s perception of their health [30]. At the same time, objective data, is important not only for its avoidance of the bias inherent in exclusively subjective scoring systems, but also for its potential to be captured continuously from a wearable sensor (such as a smartphone) from a remote location. This has enormous possibilities; one example is the ability to identify disease processes at an early stage and alert clinicians that review is required. There are some insightful examples using remote monitoring systems in the literature. For example, Huan et al. [31] devised a skin temperature remote monitoring system for the early detection of infection (error rate < 10% for detecting small temperature anomalies under 0.4 degrees Celsius) and Varatharajan et al. [32] proposed an algorithm involving foot movements which can be measured by wearable sensors which can be used to identify Alzheimer’s disease at an early stage (accuracy not reported). A recent example using a smartwatch was published by Tison et al. [33] where the Cardiogram mobile application on commercially available Apple Watches was used to identify atrial fibrillation. The device had a sensitivity and specificity of 67.7 and 67.6 respectively, which, although modest, lays the groundwork for the future management of atrial fibrillation.

A user-friendly smartphone platform may be compatible with gamification elements to improve a patient’s engagement with the application and interest in their own health. For example, Fig. 2 displays how users are presented with a graph of their SOQOL scores over time. Future iterations of the application this may be combined with success feedback or goal setting to encourage individuals to improve their health. This is particularly relevant for improving a patient’s daily step count, which accounts for 25% of SOQOL and is largely dependent on personal motivation. This is supported by Johnson et al. [34] who performed a systematic review on the effectiveness of gamification strategies as health interventions and concluded that gamification was most effective in improving physical activity levels amongst individuals (reported in 13 out of 19 studies reviewed). Importantly, clinicians when reviewing patient SOQOL scores must be aware of contextual bias where patients may falsely report better scores on the subjective component of SOQOL in an attempt to show improvement over time and achieve in-app goals.

However, this study has limitations. The normative population data for this paper was sourced predominantly from European countries which may not be representative of the rest of the world. Additionally, the Argus dataset from which we sourced normative daily step count data is biased towards mid-high income countries with access to smart devices [17]. Future studies can survey SOQOL scores in low-income countries to build a more comprehensive representation of global SOQOL averages.

In addition, the accuracy of walking speed data obtained from smartphones is at present a concern. Commercial smartphones such as the iPhone relying on distance-based calculations using GPS software demonstrate errors of up to 43% [34]. The widespread implementation of SOQOL requires a more accurate method of assessing walking speed. One solution is for future iterations of the SOQOL smartphone application to use the phone’s in-built multiaxial accelerometer to calculate walking speed. The current barrier to this is the resulting significant drain on battery life, however, this can be circumvented by operating on a threshold. For example, the device may only use the triaxial accelerometer only once a threshold of, say, 10 consecutive steps have occurred, and 10 s of activity have elapsed. This would limit usage of the accelerometer and preserve battery life, while also allowing a higher proportion of data points to be collected from longer walking bouts to represent a patient’s true average walking speed more accurately. Another limitation of the use of a smartphone to collect activity data is that the device may not be worn by the patient at all times of the day, leading to some amount of missed activity. A possible improvement for future studies could be the use of a smartwatch to collect activity metrics as these are more typically worn at all times of the day.

Further considerations when interpreting SOQOL scores are that some disease states may not necessarily lead to poorer scores on SOQOL. For example, a patient with mania may have an elevated level of physical activity and may self-report excellent scores on a questionnaire. Therefore, SOQOL scores must be interpreted in the context of the broader clinical scenario of each patient. Finally, before this score can be recommended for clinical practice, it must be examined by large-scale validation and feasibility studies.

## Conclusion

The detection and quantification of decline and recovery in physical and mental health across a broad range of pathologies and life events remains a challenge. In this preliminary study, the authors present a simple health score—SOQOL—with both subjective and objective components. While SOQOL shows potential in providing information that appears easy to measure and understand for health practitioners across various specialties, thereby assisting in healthcare decisions, it is important to note that these findings are initial and primarily suggestive. This study underscores the necessity for future, more comprehensive research to fully validate and refine the SOQOL, particularly in addressing the limitations noted in our early data. As such, readers should interpret the results with an understanding of the study's exploratory nature.

## Data Availability

All data produced by this study may be viewed upon contacting the corresponding author.
